# Technologized Reproduction in Space: A Space‐Bioethical Case for Assisted Procreation

**DOI:** 10.1111/bioe.70116

**Published:** 2026-04-29

**Authors:** Maurizio Balistreri, Konrad Szocik

**Affiliations:** ^1^ DISTU (Department of Humanities: Languages, Literature and Cultural Heritage) University of Tuscia Viterbo Italy; ^2^ Department of Social Sciences University of Information Technology and Management in Rzeszow Rzeszow Poland

**Keywords:** artificial womb, bioethics, feminism, genome editing, reproduction, space exploration

## Abstract

With the increasing feasibility of space colonization, the issue of reproduction in space is becoming more relevant. As new settlements on the Moon, Mars, and other celestial bodies emerge, ensuring generational continuity will be essential for the survival and growth of these communities. The challenges of space living, such as extreme environmental conditions and limited resources, raise critical questions about the practicality and morality of reproduction in these contexts. This paper argues that in extraterrestrial space, human reproduction based on assisted reproductive technologies is morally preferable to sexual reproduction. We explore the benefits of technologies like embryo selection, genome editing, and artificial wombs, emphasizing their potential to mitigate the risks posed by space environments. There is no doubt that the idea of human reproduction based solely on technology is controversial and counterintuitive, even if it were to be applied only, at least initially, for the continued existence of the human population settled in space. Nevertheless, there are strong rationales for the fact that non‐sexual reproduction is not only beneficial, but also has moral advantages. Finally, we also examine the social and political implications of a society that moves beyond sexual reproduction, including the reduction of gendered oppression and the redefinition of family structures. We suggest that these advancements could transform reproduction not only in space but also on Earth, potentially reducing injustices caused by human biology and reshaping societal norms and traditional view on family.

## 1 Introduction

With the intensification of space projects, the possibility of establishing new settlements on the Moon and other planets is becoming increasingly realistic [1, 2]. We imagine that the communities forming in these new settlements in space (space stations, asteroids, planets, etc.) will inevitably face the issue of reproduction [3, 4]. This matter arises not only because people living in these communities may desire to have children, but also for practical reasons tied to the survival and growth of the community itself. Ensuring generational continuity will be essential to preserving the community's identity, culture, and skills. Furthermore, the birth of new individuals could contribute to demographic growth or stabilization, facilitating the achievement of a minimum viable population. This would, in turn, reduce the risk of the community's extinction due to random events, ensuring its ability to maintain infrastructure and promote economic growth [5, 6]. Naturally, a community could also achieve self‐sufficiency by encouraging immigration or developing technologies based on artificial intelligence [7]. However, especially in the early stages of constructing new settlements on other planets, living conditions could be particularly challenging (e.g., very small shared habitats and long work shifts). Moreover, space colonization may entail one‐way migration, since genetic adaptations required for survival beyond Earth could prevent return. This could also act as a factor discouraging immigration. Finally, ensuring adequate human populations in distant colonies or extra‐solar settlements may also pose significant challenges [8]. Life on Earth may not be possible due to potential future catastrophes, so the idea of a habitat in space, as it is widely perceived in literature, usually assumes self‐sufficiency. And self‐sufficiency and independence require the succession of generations. The thesis we intend to defend is that, in extraterrestrial space, reproduction based on reproductive technologies is morally preferable to sexual reproduction (based on natural intercourse). Moreover, we will not simply argue that assisted reproduction is preferable in space; we will try to imagine what it means to live in a society that moves beyond sexual reproduction and the need to sustain the development of an embryo within a human body. We will show that, in addition to benefiting the health and well‐being of embryos, fetuses, and women, assisted reproduction could also offer significant political and social advantages. The greatest benefit could be the reduction of women's oppression caused by biological reproduction and the idea that women should be responsible for caring for children. While many women certainly value not only pregnancy and childbirth, but also motherhood itself, this does not change the fact that, both historically and for many women today, biological reproduction is a source of oppression. Men do not experience similar oppression derived from reproductive biology. The technicization of reproduction could challenge the traditional family model by decoupling reproduction from the female body, potentially questioning the viability of this family structure.

First, we will begin by discussing the difficulty for humans to engage in sexual intercourse in space, and then move on to address the challenges of producing mammalian embryos through fertilization (Section 2). We will then explain why genome editing interventions may be necessary in space (Section 3). Finally, we will analyze the societal consequences of human reproduction based solely on technology (Section 4) and the impact this extraterrestrial societal model could have on Earth (Section 5).

## 2 Is It Possible to Reproduce (Sexually) in Space?

In recent years, researchers have started to focus on (human) sexuality in space [9]. However, we still do not know if sexual intercourse in space is actually possible [10, 11, 12, 13]. In microgravity, elastic straps or other fastening methods would be necessary to prevent bodies from floating apart [14]. Moreover, in the absence or reduction of gravity, astronauts may have more difficulty in achieving an erection, as blood pressure and testosterone levels tend to be lower [15]. Even if sexual intercourse were possible, fertilization might still prove challenging, as microgravity can negatively affect spermatogenesis [16] and promote the degeneration of seminiferous tubules in the testes. This could lead to a significant reduction in sperm count [17]. For female astronauts, space travel and microgravity may cause disruptions or cessation of menstrual cycles, as well as reduced pregnancy rates and neonatal survival [18]. Additional complications could stem from prolonged exposure to cosmic radiation, which can accelerate the decline of the follicular pool and ultimately result in premature ovarian failure. Finally, in space, the absence (or reduction) of gravity could affect other reproductive processes, such as the ability of sperm to orient and move in the right direction within the body to reach and fertilize the egg [14].

Currently, we do not yet know whether mammalian fertilization is possible in space. So far, mammalian fertilization has only been achieved under simulated microgravity conditions, with subsequent births of animal litters reported in these cases [17]. Full developmental capability has also been observed in rat embryos that had previously spent some time in space in the Earth's lower orbit [19]. In these cases, a high mortality rate was recorded during the first week, but the surviving animals showed no health issues and were able to conceive and produce offspring. However, under non‐simulated microgravity conditions, mammalian fertilization has not yet been achieved.

The reasons why mammalian fertilization has not yet succeeded in space may be linked to both exposure to cosmic radiation and microgravity conditions. Despite progress made by researchers who have managed to cultivate mouse embryos in low Earth orbit for several days, we are still far from achieving successful births in space [20, 21, 22]. Even more distant seems the possibility of completing the entire human reproduction process beyond Earth – a goal presenting immense scientific and technological challenges. Moreover, no experiments involving human embryos or gametes brought from Earth and fertilized in space have yet been conducted in the Earth's lower orbit. The only studies conducted in space on human gametes have focused on their motility under low Earth orbit conditions [23]. Egbert Edelbroek, CEO of SpaceBorn, has expressed intentions to proceed with conception and embryo development in space, starting with mice and later using human gametes [24]. According to Edelbroek, tackling the issue of space reproduction is a necessary step for any serious extraterrestrial settlement project [25]. However, such projects remain far from realization, and the moral issues involved are enormous.

If the difficulties in achieving mammalian embryo fertilization in space are confirmed, the only way for the human species to reproduce in space would be through assisted reproduction using human embryos produced on Earth and then transferred. (If fertilization proves impossible, embryos cannot be produced in space.) However, even if fertilization were possible, it must be considered that radiation and microgravity could cause modifications in gametes, which might be passed on to children born as a result. We still do not know whether staying in space affects human gametes. In a study by Matsumura et al. [26], mice that stayed aboard the International Space Station (ISS) for 35 days showed no negative signs regarding sperm quality or the vitality of their offspring upon their return to Earth. However, in a more recent analogous study by Yoshida et al. [27], researchers reached different conclusions and observed significant changes in mouse sperm. Among these, the most critical alterations affected the hypothalamic‐pituitary‐gonadal axis, which regulates the production of sex hormones and fertility (potentially affecting erection and sperm quality). No negative effects were observed on sperm kept frozen aboard the ISS for 5 years and 10 months and later used on Earth for reproductive purposes [19]. Moreover, it appears that oocytes are capable of correcting potential damage to the genetic code of sperm caused by radiation exposure [16].

However, these studies were conducted on the International Space Station (ISS), which is located in the Earth's lower orbit, where the intensity of cosmic radiation is higher than on Earth but still lower than what would be experienced during more distant space missions (e.g., to Mars). It must be considered that the farther one moves from the Earth's lower orbit, as in projects for the settlement of the Moon and Mars, the greater the risk of damage or mutations to gametes, as astronauts would be in environments with significantly higher levels of radiation. Additionally, in these space missions, the astronauts' stay in space would in all likelihood be much longer than the 35 days studied with gametes, further increasing the risks. In the context of space travel beyond the Earth's lower orbit, it is evident that the use of technology would allow greater protection for gametes used in reproduction. Instead of reproducing sexually, individuals participating in space missions who wish to have children could use donor gametes or their own, previously collected and cryopreserved on Earth before departure.

## 3 In Space, Genome Editing Interventions Might Be Necessary

With in vitro reproduction, not only would there be greater control over the embryos produced, but it would also be much easier to perform genome editing interventions on the germline. It has been argued that having a healthy child does not necessarily require genome editing; it would suffice to select the gametes used in reproduction. Additionally, selecting not only the gametes but also the best embryo among those produced would further reduce the risk of transmitting genetic anomalies to the child [28, 29, 30]. However, couples resorting to assisted reproduction do not always manage to produce more than one embryo or even a single healthy embryo among those they eventually obtain. In space, moreover, it may be necessary to correct potential genetic anomalies in astronauts' gametes caused by cosmic radiation. Even if gametes (or embryos) imported from Earth were used in reproduction, there is always the possibility that they might sustain significant damage during the journey. Furthermore, genetic modification could prove useful for optimizing embryonic development, as space environmental conditions may make it less secure than on Earth. For example, space could disrupt the normal development of the brain (neural cells), compromising its structure and functions [19]. Additionally, embryonic development might occur at an accelerated rate, increasing the risk of genetic damage and mutations that could lead to diseases such as cancer [22]. Finally, even if fertilization and subsequent embryonic development were to proceed without issues in space, genome‐editing interventions might be necessary to improve the genetic code of individuals born there, making them better suited to live in otherwise hostile extraterrestrial environments.

If genetic modifications are deemed necessary for any space travel involving prolonged stays beyond the Earth's lower orbit, astronauts embarking on such missions should refrain from reproducing sexually. After all, genome editing interventions on the germline may not be easily practicable in vivo on embryos conceived sexually or already in the womb. It is true that genome editing experiments on animal embryos within the maternal uterus are beginning to show promising results, suggesting potential clinical applications in the future [31, 32, 33]. However, it remains uncertain whether such interventions will be safe and effective on a clinical scale. (Until now, apart from the experiments, genome editing on human or mammalian embryos has been conducted only “in vitro” as part of assisted reproduction procedures).

Even if genome editing interventions could also be applied *in vivo*, interventions on in‐vitro embryos would still be more advantageous for both the child to be born and the parents. Performing a modification (whether for therapeutic or enhancement purposes) on an embryo appears to be significantly easier if the embryo is produced in vitro rather than through sexual reproduction. Indeed, in the case of in‐vivo treatments, the effectiveness of the modifications could be influenced by the physiology of the human body, which might hinder the success of the intervention. Moreover, if the embryo is produced in vitro, the genetic modification can be performed immediately, on the fertilized oocyte or the embryo at its earliest developmental stage. On the other hand, if the embryo is produced sexually (in vivo), it would likely be at a more advanced stage of development, consisting of a significantly larger number of cells. Additionally, genome editing on embryos in vitro passes modifications to future generations, while in vivo interventions affect only one generation and must be repeated, as they target somatic rather than germline cells [34, 35]. In principle, fetal gene therapy could also target primordial germ cells, although this would be technically possible but highly challenging to achieve.^1^

Finally, even if genetic modification could be performed in vivo without complications and with the same efficacy as in‐vitro genome editing, performing the procedure on embryos in vitro might still be safer for women. Genetic interventions on the in‐vivo embryo could be significantly more invasive for women, thereby posing greater risks to their well‐being. This issue arises regardless of where such interventions are carried out; however, in a space context, one must also consider that in an emergency (such as when a woman requires medical assistance or surgical intervention), providing adequate care would be far more difficult due to operational challenges and resource scarcity. Even if the genome editing procedure posed no direct problems, the series of preliminary checks and examinations could still be burdensome and potentially carry risks. These procedures might increase the likelihood of complications during pregnancy, such as infections or adverse reactions associated with the manipulation and monitoring of the embryo. Furthermore, if the fetus is inside the mother's body (as opposed to outside, as with in‐vitro interventions), there is a risk that the perception of the fetus as a patient might lead to a devaluation of the woman's interests [34, 36] and an expectation that she should do everything possible to improve the fetus's condition [37].

Moreover, space does not seem to be an ideal environment for sexual reproduction, as it necessarily involves pregnancy and childbirth. On the other hand, an artificial womb (ectogenesis) would significantly reduce the health risks and challenges faced by women participating in space missions. Microgravity, for instance, disrupts the normal functioning of bodily fluids, potentially leading to increased intracranial pressure and changes in blood pressure [38, 39].

## 4 The Consequences of Reproduction Based Solely on Technology

Assuming that pronatalism is the dominant position on Earth, it can be assumed that reproduction based solely on technology will avoid replicating the culture of pronatalism in space. Pronatalism assumes that procreation is not only something good, but also something recommended, advisable and even necessary. If we accept that the ultimate goal of human presence in the cosmos is permanent settlement driven also by the desire to save the duration of the human species and potentially other species as well [40], then consequently, at least to some extent, any permanent human habitat in the cosmos must also be pronatalist, because it must secure the succession of generations, especially in a situation where the space settlement would be independent, self‐sufficient, and migration from Earth would not be possible. However, this model of procreative ethics, if it were to be applied to humanity's future in space, could cause negative moral consequences. Pronatalist ethics, culture, and politics of space missions may lead to the fact that in the process of selecting candidates for missions, as well as planning the organization of life in space, issues of sexual life, dating and mating plans will become important, and procreative goals will become overriding objectives. Consequently, procreation will become an important part of public life, with possible oppression toward procreation and a possible temporary ban on procreation depending on political expectations and the demographics.

If reproduction in space settlement is based solely on technology in such a way that birth may no longer involve the body as embryos develop in an artificial womb, the main cause of women's oppression on Earth will disappear. That cause was precisely women's reproductive biology. From the fact of being pregnant and bearing children, the concept of women's duty to take care of them exclusively was derived [41]. If women are not needed to carry pregnancies and give birth, the stereotype of women as predestined to take care of children and designated to the private sphere will lose its main justification [42]. This is not the only cause of oppression of women, but it is one of the most troublesome and widespread.

If in our thinking about the future in space we adopt a feminist perspective exposing unjust power structures, mechanisms of oppression and discrimination, then planning a new society in space free from the discrimination and inequality known on Earth will also require concern for gender equality [1, 43]. Is it possible where women bear children and are expected to maintain a special bond with their children? To avoid replicating in space the oppression of women associated with a specific understanding of their reproductive biology, it is desirable to create a reproduction based only on technology. Ectogenesis will certainly reduce or eliminate the influence of procreation policies on the selection of candidates for space missions, as well as reduce the perception of women through the prism of their procreative utility, and spare women the health problems and life‐threatening risks that can be caused by pregnancy and childbirth in space [44].

This is required especially in such a specific environment as the space environment, which, as an extreme environment, fosters a belief in moral exceptionalism that can justify the lowering of moral norms, assuming their transitory nature only. It cannot be guaranteed that technologically mediated reproduction will prevent the emergence of some form of oppression of women, since there remains the problem of offspring care, as well as care in general, which on Earth is ceded almost exclusively to women [45]. Depending on the strength of ties to Earth, and especially the strength of the influence of traditions and habits known on Earth on people living in the cosmic habitat, the motif of women as caregivers may be present. However, if reproduction is realized outside the woman's body, the justification for delegating women to this care should diminish significantly.

Another consequence of the technicization of human reproduction will be the impact exerted on the understanding of the family. The Western world is dominated by the nuclear family model, which is considered by Marxist and socialist feminism to be a necessary condition for the persistence of patriarchy and capitalism [46]. In this model, women are delegated to bear children and care for them, while men are responsible for supporting the family and maintaining social and political order. Bringing more and more new people into the world, who are needed under patriarchy and capitalism to serve as laborers, workers, customers, taxpayers, soldiers, plays an important role in this order [47]. Consequently, women's work in the public sphere is not as important (this one should be provided only or almost exclusively by men) as their reproductive work, both biological and social. If biological reproduction is decoupled from the female body, this will lead to a weakening and perhaps even questioning of the viability of maintaining the nuclear family model.

Even if the nuclear family model were to persist, perhaps the negative consequences of this model in terms of the primacy of one's own genetic offspring over the rest of society would diminish. One of the negative consequences of this primacy is the drive to accumulate resources for one's own genetic offspring [48]. Reproduction through technology could weaken the negative consequences of attachment to one's own biological offspring, which result in the aforementioned, often irrational, pursuit of resource accumulation exclusively for one's own children. The new type of reproduction applied to space may force a shift from a biological to a sociological model of ties between offspring caregivers and offspring [49].

Reproduction carried out solely by technology can reduce the fertility rate. The high fertility rate on Earth is caused largely by the culture of pronatalism, which is present in religion, ethics, politics, as well as popular among the public and transmitted in education. It leads to the belief that not having children is wrong, generating pressure to reproduce. Separating reproduction from biology will therefore lead to the elimination of this pronatalist pressure. Reproduction in space may be more rational, implemented for the sake of maintaining the continuity of the human population, but nothing more than that. Reproduction on Earth is often irrational, leading to problems for the parents, the children born, and the rest of society. It also leads to more and more resource exploitation and environmental pollution [50]. It can be assumed that reproduction in space will have to be regulated due to resource and infrastructural capabilities. However, it is possible to imagine a scenario in which technological adaptation to life in space will be so efficient that the production of more and more new people will not be a problem. However, it will cause environmental pollution and resource exploitation just as it causes on Earth. From this point of view, it is more optimal to maintain a sufficiently small number of people, which may be possible through reproduction by means of technology.

The consequences outlined above in the form of a reduction in pronatalist oppression, a potential reduction in fertility rates, as well as a possible change in the family model will also influence a change in thinking about child adoption, as well as the concept of multiparenting. The weakening of the specific sense of connection produced by biological reproduction, as well as the weakening of the belief that it is the only appropriate way, may change the way potential parents think about children who are not biologically related. It may also increase the attractiveness of multiparenting, in which a child has more than two parents [51].

## 5 What Could Be the Consequences of This Scenario for the Human Population on Earth?

Futures studies consider scenarios that are possible, probable and preferred [2]. The discussed scenario of reproduction mediated by technology in future space settlement is both possible and probable. But is this the preferred scenario we should be aiming for? If so, reproduction based solely on technology could be introduced on Earth first, without waiting to obtain special justification for the harsh conditions of the space environment. Purely technology‐based reproduction in space would then only be an extension of the solution used on Earth. If this were the case, appropriate measures directed at the transition from biological reproduction to technological reproduction would already have to be taken, regardless of the possibility of its potential use in space.

Even if we assume that this is the preferred scenario not only in space, but also on Earth, it seems that the only way to implement it is to prepare a technology‐only reproduction option for human habitat in space. As with the concept of radical human enhancement involving moral bioenhancement, space is the optimal place to test these radical, controversial technologies. From the point of view of the morality of biomedical procedures, this is not ideal, as it may lead to a situation of moral exceptionalism, which we criticize, stressing that there should be one morality on Earth as well as in space [2] This is also in line with the perspective of feminist bioethics of space exploration, which points out that the same action judged immoral on Earth is also so in space, and vice versa [1]. Thus, when we talk about the attractiveness of space as a place for testing controversial solutions, we mean not so much their moral controversiality, but their contradiction with intuitions commonly shared by people. In this context, the notion of space as a particularly challenging and dangerous place may provide reinforcement to justify the use of controversial new solutions.

There are several strong arguments that in a more realistic scenario, in which technology‐based reproduction is introduced in space first, the population remaining on Earth will be inspired by the population living in space to abandon sexual reproduction. Arguments in favor of such a scenario refer to the status of women and gender nonbinary people, climate change and environmental degradation, and the attractiveness of life in space.

If the use of technology‐only reproduction in space were to significantly reduce the oppression of women in accordance with the dynamics described above, the knowledge of this transmitted to the human population remaining on Earth could encourage the abandonment of sexual reproduction. We assume here that pronatalist procreative oppression will continue into the future. The shift to technology‐only reproduction may be attractive not only to women, but also to gender nonbinary people, as well as to anyone who feels pressure to procreate, including men. Non‐heteronormative people who are deprived of the right to have children because of their non‐heteronormativity may see in the concept of non‐sexual reproduction the possibility of having offspring regardless of the obstacles that are now placed in front of them in most of the world because of their sexual orientation [52]. Assuming that humanity will develop in a linear fashion with respect to technological progress, we can assume that digital health technologies, including for pregnancy, will be continually developed and implemented. A negative consequence is the intensification of monitoring, controlling, and therefore also disciplining the female body and women's decisions. Technological monitoring is driven by a morally legitimate concern for the health of the embryo and fetus, but the side effect is to limit the autonomy and choices of the pregnant woman [53]. As a result of digital health technology, her pregnancy becomes a public matter in an even more specific sense. In the case of the artificial womb, the condition of the embryo and fetus will probably be monitored to an even greater extent, but this will not be at the expense of a woman's freedom, autonomy and comfort.

Another rationale that may convince the Earth's population to abandon sexual reproduction is the environmental and climate change context. Climate change is particularly dangerous for pregnant women [54]. It can be expected that the artificial womb will be a safe and stable environment for the embryo and fetus, in contrast to the deteriorating living conditions of many women, especially the poorer ones, most severely affected by climate change and environmental degradation. In this sense, asexual reproduction may be a legitimate alternative to protect both mothers and future children, which is expected for ectogenesis used in space, for example, in sheltering below the surface to better protect against cosmic radiation [44]. The reference to deteriorating living conditions caused by climate change, combined with the awareness of the possibility of realizing reproduction through technological means, can also reduce the tendency to biological procreation and bring new people to live in increasingly difficult environmental conditions. Then procreation by technological means would be realized only in very justified situations and would not involve forcing women or men.

Another rationale in favor of abandoning biological reproduction by the population remaining on Earth may be the attractiveness of life in space. We assume that in humanity's future scenario, in which the human population lives and reproduces in a space habitat, this life must meet minimum conditions for quality of life and remain attractive. As we have noted, initial conditions may be particularly harsh and discourage immigration; however, over time, circumstances could change radically and reverse the situation, potentially encouraging migration. The attractiveness of life in space may encourage those remaining on Earth to abandon biological reproduction in favor of non‐sexual reproduction. Depending on the family and child‐rearing models available, sexual reproduction may no longer be seen as attractive and justified, especially for those who intend to move from Earth to a settlement in space.

In summary, the counterintuitive idea of technology‐mediated reproduction, if it were widespread, and perhaps the only option used in space, could become attractive to the population remaining on Earth for several interrelated reasons. At their core is the oppression of women caused by pronatalism. The possibility of decoupling reproduction from the female body would free women from many of the expectations and obligations formulated by society regarding the concept of women as mothers and caregivers [55]. Women who are interested in having offspring and have the ability to use the artificial womb may choose to reproduce through technology. The increasingly onerous effects of climate change, particularly felt by women, may discourage at least some women from engaging in biological reproduction, making the concept of having children more grounded. Finally, the possibility of participating in a space exploration project and the hope of settling in space—assuming it is an alternative that is attractive to those living on Earth—may prompt both men and women to abandon biological reproduction in favor of reproduction through technology. Having a child will cease to be an activity determined by the prospective parents' biology, age, or physical potential, which could help reduce the time pressure to have children within a certain time frame. Thanks to technological procreation, having children for those individuals for whom having their own offspring is an essential part of their life plan can be postponed until what is deemed the most optimal moment. Understood in this way, non‐sexual reproduction obtains a strong moral justification and is morally superior to biological reproduction, which has a number of negative side effects, especially for women experiencing procreative oppression.

## 6 Conclusion

There is no doubt that the idea of human reproduction based solely on technology is controversial and counterintuitive, even if it were to be applied only, at least initially, for the continued existence of the human population settled in space. Nevertheless, there are strong rationales for the fact that non‐sexual reproduction is not only beneficial, but also has moral advantages over biological reproduction. In addition to the physiological and health benefits associated with greater protection of embryos, fetuses, and pregnant people in space, the moral advantage of this type of reproduction is also evident in the social and political dimensions, namely the reduction of oppression caused by biological reproduction on Earth.

## Data Availability

The authors have nothing to report.

## Endnotes

1 We thank one of Reviewers for bringing this issue to our attention.
